# Few-Photon Spectral Confocal Microscopy for Cell Imaging Using Superconducting Transition Edge Sensor

**DOI:** 10.3389/fbioe.2021.789709

**Published:** 2021-12-15

**Authors:** Kazuki Niwa, Kaori Hattori, Daiji Fukuda

**Affiliations:** ^1^ Research Institute for Physical Measurement, National Metrology Institute of Japan, National Institute of Advanced Industrial Science and Technology (AIST), Tokyo, Japan; ^2^ AIST-UTokyo Advanced Operando-Measurement Technology Open Innovation Laboratory, Kashiwa, Japan

**Keywords:** fluorescence cell imaging, superconducting transition edge sensor, confocal microscope, spectral imaging, photon counting

## Abstract

A superconducting transition edge sensor (TES) is an energy-dispersive single-photon detector that distinguishes the wavelength of each incident photon from visible to near-infrared (NIR) without using spectral dispersive elements. Here, we introduce an application of the TES technique for confocal laser scanning microscopy (CLSM) as proof of our concept of ultra-sensitive and wide-band wavelength range color imaging for biological samples. As a reference sample for wide-band observation, a fixed fluorescence-labeled cell sample stained with three different color dyes was observed using our TES-based CLSM method. The three different dyes were simultaneously excited by irradiating 405 and 488 nm lasers, which were coupled using an optical fiber combiner. Even when irradiated at low powers of 80 and 120 nW with the 405 and 488 nm lasers respectively, emission signals were spectrally detected by the TES and categorized into four wavelength bands: up to 500 nm (blue), from 500 to 600 nm (green), from 600 to 800 nm (red), and from 800 to 1,200 nm (NIR). Using a single scan, an RGB color image and an NIR image of the fluorescent cell sample were successfully captured with tens of photon signals in a 40 ms exposure time for each pixel. This result demonstrates that TES is a useful wide-band spectral photon detector in the field of life sciences.

## Introduction

Spectral imaging provides information about the distribution of multiple molecules and other entities via spectral analysis. In the field of life sciences, spectral confocal laser scanning microscopy (CLSM) provides substantial information on the cellular dynamics associated with various biomolecules.

In CLSM cell-imaging applications, samples are labeled with fluorescent dyes and irradiated by excitation light to obtain fluorescence signals. However, in living cells, excitation light irradiation deteriorates cells from their native state and causes photobleaching of fluorescent dyes (Icha et al., 2017). Therefore, it is ideal to minimize excitation light irradiation. However, a decrease in the excitation light results in a decrease in the fluorescence signal. In the case of auto-fluorescence, which is emitted from endogenous fluorescent molecules in living organisms, signals are weak even when the molecules are irradiated with high-power excitation light. Moreover, there is an increasing demand for a highly sensitive photodetector, which has a smaller background noise and higher detection efficiency, to observe fluorescence signals from cellular samples at the few-photon signal level.

Consequently, numerous efforts have been made to develop more sensitive photodetectors. A transition-edge sensor (TES) is a superconducting film that acts as a thermometer and can measure weak energy at the level of a single photon (Irwin and Hilton, 2005). As the energy of a single photon is related to its wavelength by the following equation, the TES directly distinguishes the energy of an absorbed photon without using any spectral element, after which the wavelength of the photon is distinguished.

$$E=\frac{\mathrm{hc}}{\lambda }$$

Thus, a TES can be used as an ultrasensitive spectral photodetector. The spectral efficiency of a TES is determined by its absorption/reflection properties. We designed and constructed a TES to absorb a wide range of wavelengths of photons from the visible to near infrared (NIR) range using an optical cavity structure (Fukuda et al., 2011).

Previously, we reported a concept proof of the TES technique for ultra-sensitive and wide-band spectral imaging (Niwa et al., 2017). Using a three-color ink printing test pattern (a photograph), RGB color images and NIR images were successfully captured under epi- and side-illuminated conditions. Using a TES in a 50 ms exposure time for each pixel, tens or fewer photon signals were detected to provide a color image in a single scan. Although a few photon signals for each pixel were sufficient to provide RGB color images, more signals were required to generate the wavelength spectral data. Therefore, spectral data at a single pixel were obtained by increasing the exposure time to 200 s. These results suggest the high potential of TES for use as a spectral photon-counting detector in CLSM for biological microscopy applications.

For cell imaging applications, we constructed CLSM optics using TES as a photodetector (Fukuda et al., 2017). Using microscopy optics, preliminary observation of fluorescent-labeled cell samples was performed using a single excitation laser (488 nm) to excite two fluorescent dyes (Alexa 488 and MitoTracker). Although two dyes were detected, the cells were labeled with three dyes, and full-color imaging was needed to obtain signals from all three fluorescent dyes. Because a TES is sensitive to a wide wavelength range of photons, more fluorescent dyes are detected simultaneously, which provides RGB full-color images. In this report, we introduce an improvement in CLSM optics to excite a wider range of fluorescent dyes and present the results of an extended investigation of cell samples. The excitation laser irradiation and photon counting signals were investigated to consider full-color image caption sensitivity at the photon-counting level by the TES.

## Materials and Methods

### Microscope Optics

**FIGURE 1:**
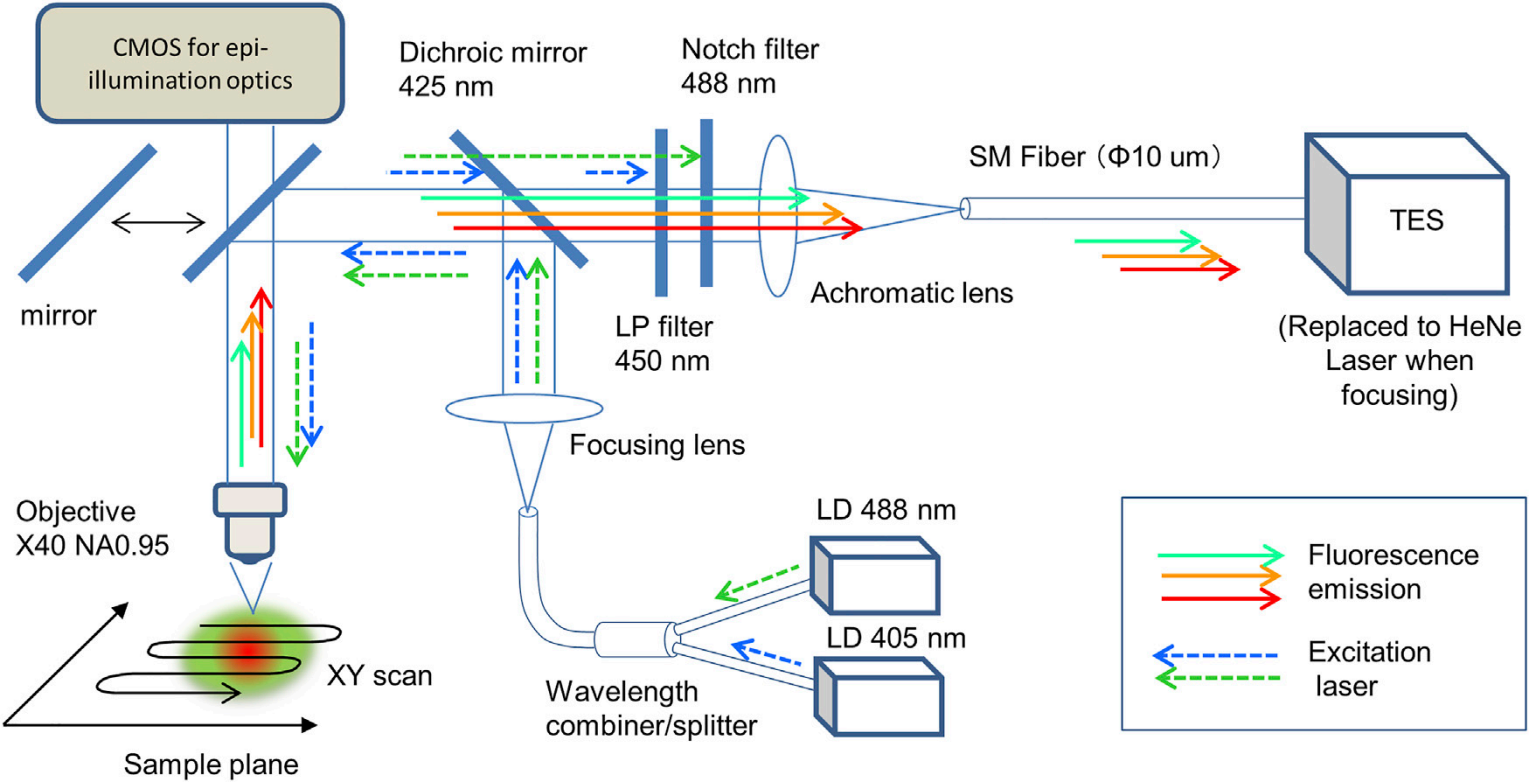
Confocal laser microscope optics with a fiber-coupled TES. Two excitation lasers at 405 and 488 nm were combined using a fiber combiner and focused on the sample plane under the objective (×40 NA0.95). Collimated fluorescence photons were focused on the core (10 μm diameter) at the end of the optical fiber, then introduced into the TES. The sample on the focal plane was scanned to accumulate an imaging data.

The design and construction of a scanning microscope system using a TES as a photodetector with an optical fiber has been reported (Niwa et al., 2017), and confocal optics with a single excitation laser at 488 nm have been employed for CLSM (Fukuda et al., 2018). Additionally, for full-color cell imaging, previously developed microscope optics were modified here (Figure 1). To irradiate with two distinct lasers for simultaneous excitation of three fluorescent dyes, 405 and 488 nm pig-tailed optical fiber diode lasers (LP405-SF15 and LP488-SF20, Thorlabs, USA) were combined using a wavelength combiner/splitter (GB29F1, Thorlabs, USA). For fluorescent signal detection, a dichroic mirror of 425 nm (DMPL425, Thorlabs, United States), a long pass filter (FELH450, Thorlabs, USA), and a notch filter (NF488-15, Thorlabs, USA) were employed. The magnitude and numerical aperture (NA) of the objective (UPLXAPO × 40, Olympus, Japan) were 40 and 0.95, respectively. A prepared microscope slide (FluoCells Prepared Slide #1, Tremo Fisher Scientific, USA) was used as a test sample for cell imaging. The test sample was mechanically scanned using a motorized XY stage (BIOS-105S, Optosigma, Japan) under a microscope. Stage control, data accumulation, and image processing were performed using LabVIEW software (National Instruments, USA).

To adjust and focus the optics, a He–Ne laser (Melles Griot, United States), epi-illumination optics (omitted in Figure 1, Olympus, Japan), and a CMOS camera (Infinity 1–2C, Lumenera Corp, Canada) were used. To compare the detection efficiency of the proposed TES with that of the conventional method, a photomultiplier tube (PMT; H18010 Hamamatsu Photonics, Japan) was used instead of a TES, where a 532 nm laser diode (DJ532-10, Thorlabs, USA) were added to the optics (Supplementary Figure S1). The irradiated excitation laser power (W) at the focal plane was measured using a microscope slide-type power meter (S170C, Thorlabs, USA), which was calibrated for each wavelength.

### TES Single-Photon Spectral Detector

The TES used as the photodetector in confocal microscopy is a fiber-coupled TiAu-based optical TES (Fukuda et al., 2011). The energy of a single photon absorbed by the TES was converted into the increase in electric resistance, followed by measurement of the voltage change using a superconducting quantum interference device (SQUID) amplifier associated with the TES. The TES and SQUID amplifiers were placed on a cold stage in an adiabatic demagnetization refrigerator, where the temperature was stabilized at 100 mK. To obtain the wavelength value from the measured energy of the incident photons, the pulse signal height of the SQUID voltage was calibrated using a 1524-nm pulse laser (PLP-10-155, Hamamatsu, Japan) to irradiate with multiplexed photons with energies of 762 nm (*n* = 2), 506 nm (*n* = 3), and 381 nm (*n* = 4).

To depict a color image, the photon signals detected by using the TES were distinguished by their wavelength as follows: photons shorter than 500 nm were blue, while those of from 500 to 600 nm were green, from 600 to 800 nm were red, and from 800 to 1,200 nm were in the NIR range. Additional details concerning optical photon detection via TES have been described elsewhere (Hattori et al., 2018).

## Results

### Fluorescent Cell Imaging Using a TES

**FIGURE 2:**
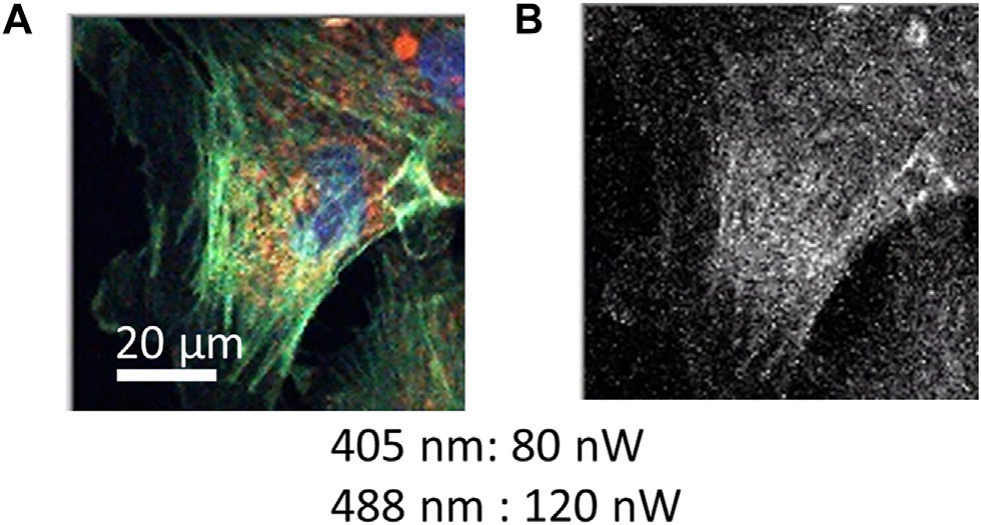
Color image obtained by a confocal scanning microscope using a TES. A fixed cell sample stained with MitoTracker (red fluorescence for mitochondria), Alexa 488 (green fluorescence for F-actin), and DAPI (blue fluorescence for nuclei). Scanning data of 200 × 200 pixels (0.4 μm for each pixel step) is depicted as **(A)** RGB color and as **(B)** NIR images, which were obtained simultaneously. Irradiation power of excitation lasers is indicated. Exposure time of one pixel was 40 ms, and the total duration was 66 min.

Because TES is an energy-dispersive photodetector, a wide wavelength range of photons from visible to NIR was simultaneously detected with the wavelength information. Each photon signal was distinguished into blue, green, red, and NIR in accordance with the wavelength. As a test target, a prepared microscope slide containing bovine pulmonary artery endothelial cells (BPAECs) was observed using a TES. The BPAECs in the slides were stained with MitoTracker Red (MitoTracker) for mitochondria, Alexa Fluor 488 (Alexa 488) for F-actin, and 4′,6-diamidino-2-phenylindole (DAPI) for nuclei. The excitation laser power at the focal plane measured using a power meter were 80 and 120 nW for the 405 and 488 nm lasers, respectively. Color images of the BPAEC were successfully constructed from the datasets of red for mitochondria, green for F-acting, and blue for nuclei (Figure 2A). Simultaneously, photon signals categorized as NIR provided an NIR image (Figure 2B). It took 66 min to scan 200 × 200 pixels with 40 ms exposure time for each pixel. The scanning step of each pixel was 0.4 μm. Photon counting signal intensities in the presence and absence of a cell in the images were analyzed (Supplementary Figure S2).

### Fluorescent Cell Imaging Using a PMT

**FIGURE 3:**
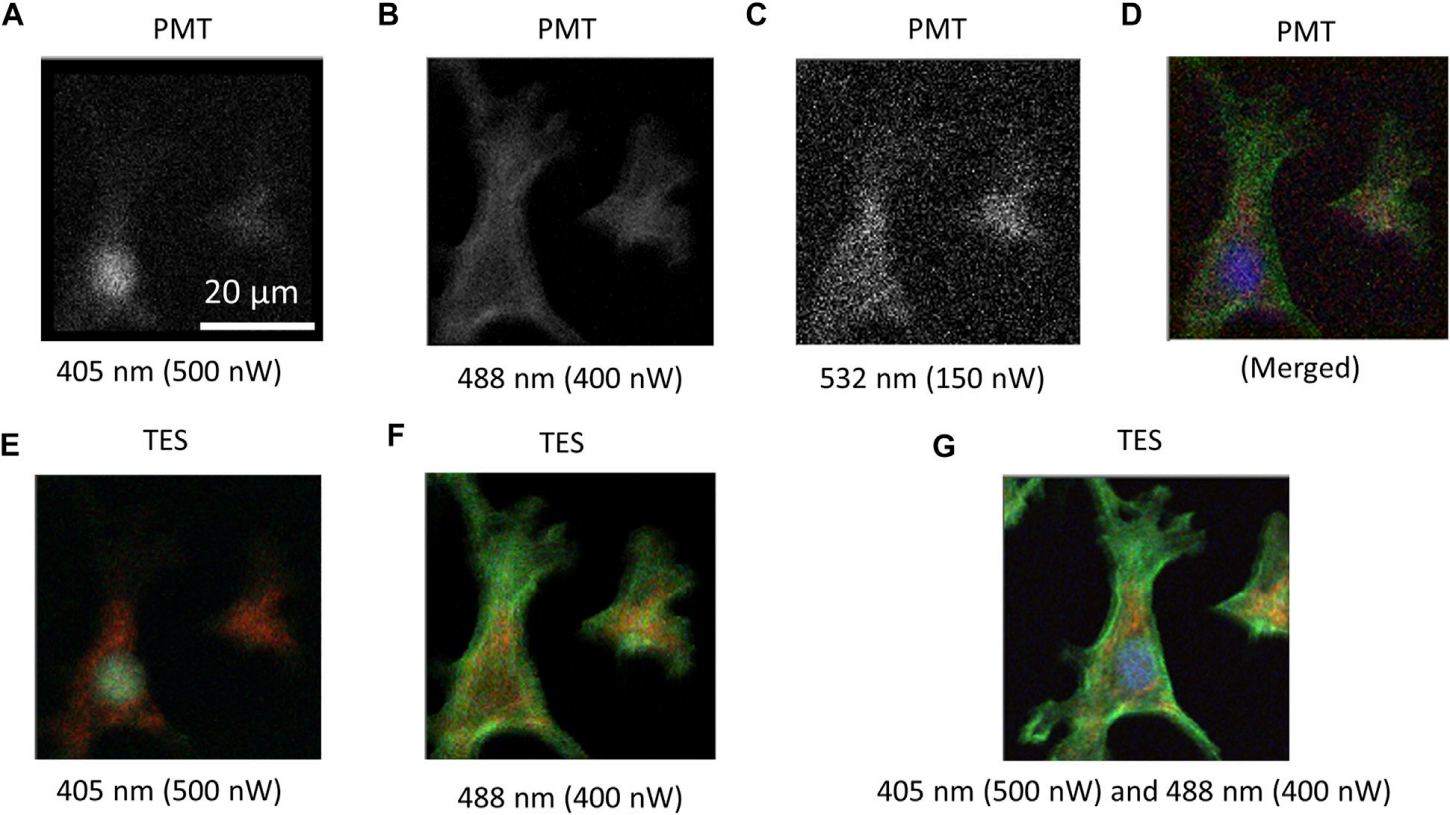
Images obtained by confocal scanning microscopy using PMT and TES. When using PMT, scanning data were collected to provide a grayscale image individually for each fluorescent dye. Grayscale images captured using PMT for **(A)** DAPI excited by 500 nW of 405 nm laser, **(B)** Alexa488 by 400 nW of 488 nm laser, **(C)** MitoTracker by 150 nW of 532 nm laser. **(D)** Pseudo-color image developed by digitally merging the three grayscale images. **(E)** RGB color image captured using a TES under the same irradiation and scanning condition as **(A)**, where mitochondria were detected in red color and distinguished from nuclei. **(F)** RGB color image captured using a TES in the same condition as **(B)**, where mitochondria were also detected and distinguished from F-actin. **(G)** RGB color image captured using a TES by irradiating the cell with excitation lasers of 405 and 488 nm at the same power as using PMT. A single scan was sufficient to create an RGB image of all dyes by 405 nm laser and 488 nm laser. Here, a 532 nm laser could be omitted. Scanning conditions were 200 × 200 pixels, 0.25 μm scanning step, and 20 ms exposure time for all images. The total duration was 48 min.

**FIGURE 4:**
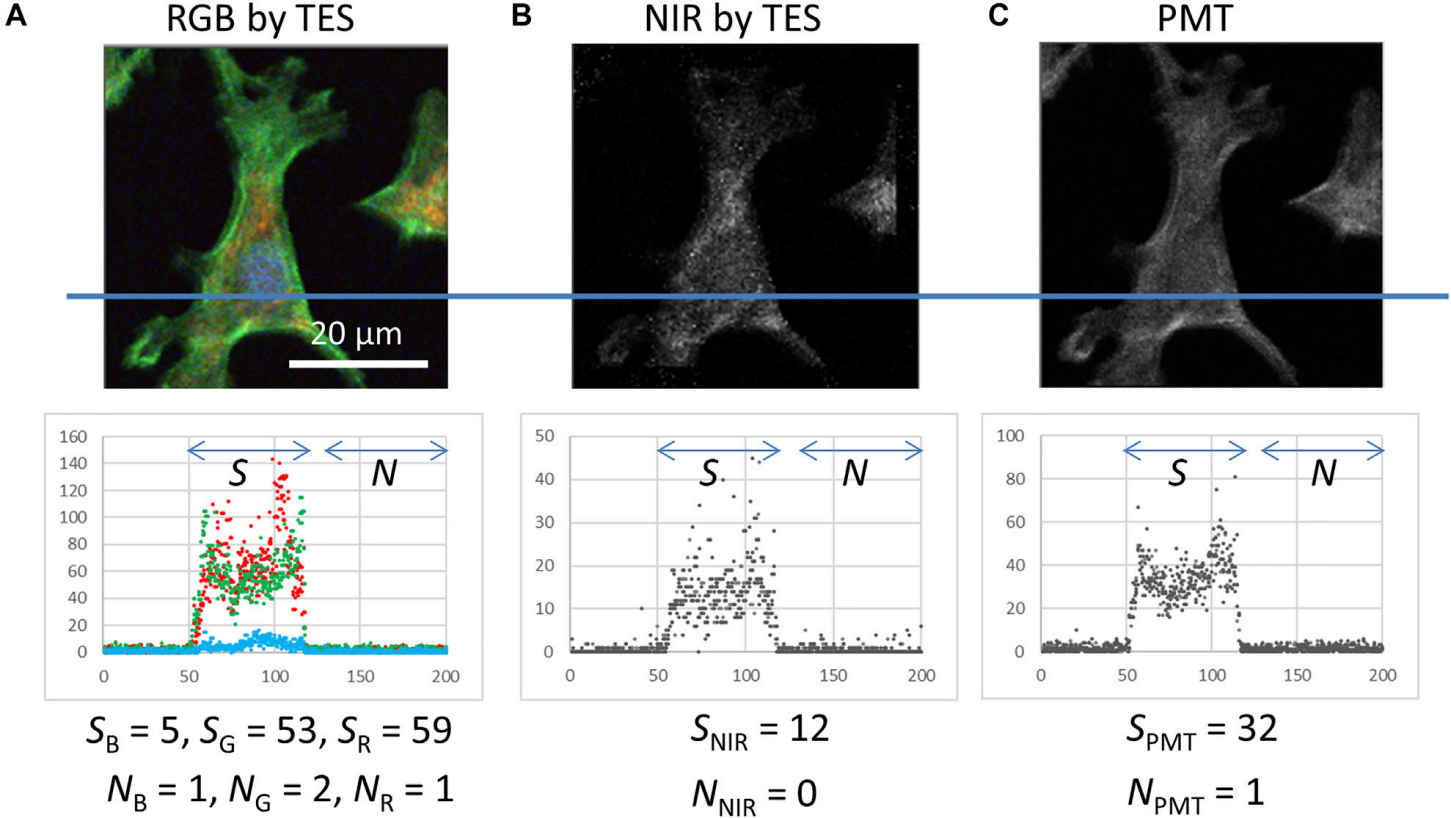
Histograms of photon counting signals detected using the TES and PMT at the transection lines (including five pixels width) on the images and signal (*S*) and background noise (*N*) intensity as medians of the area indicated by arrows. **(A)** RGB image obtained using the TES, which is identical to the data in Figure 3G. **(B)** NIR image obtained using the TES, which was simultaneously captured beside **(A,C)** Grayscale image obtained using the PMT. These data were captured under the same excitation laser irradiation (500 and 400 nW for 405 and 488 nm lasers, respectively) and scanning (200 × 200 pixels, 0.25 μm step, and 20 ms exposure time) conditions.

For comparison, CLSM was constructed using a PMT. Cell images of the test slide of the BPAEC were captured by irradiating the cells with excitation lasers one by one (Figures 3A–C). To excite DAPI for nuclei imaging, the cells were irradiated with 500 nW of a 405 nm laser (Figure 3A), Alexa 488 for F-actin was irradiated with 400 nW of a 488 nm laser (Figure 3B), and MitoTracker for mitochondria was irradiated with 150 nW of the 532 nm laser (Figure 3C). A pseudo-color image was obtained by merging the three independent images (Figure 3D). Using the TES, RGB color images were captured by a single scan at the same irradiation power, as shown in Figure 3A (Figure 3E) and Figure 3B (Figure 3F). Although a 532 nm laser was not irradiated to excite MitoTracker, a red-colored signal was observed. Hence, by combining 405 and 488 nm lasers, RGB color images were obtained at once. In all cases using both PMT and TES, it took 48 min to obtain a single image by scanning 200 × 200 pixels with 20 ms exposure time for each pixel. The scanning step of each pixel was 0.25 μm. Photon counting signal intensities from the presence and absence of a cell in the images were analyzed from the cell images obtained using TES and PMT under the same irradiation and scanning conditions (Figure 4).

## Discussion

### Cell Imaging *Via* CLSM Using TES

The step size for each pixel in Figure 2 was 0.4 μm. Under these conditions, internal cellular structures of actin filaments (green) and mitochondria (red) were successfully imaged, indicating that the optical system of the confocal microscope was constructed correctly (Figure 2A). Because the background signals from the empty area in the images were as small as 0–2 counts (Supplementary Figure S2A), a clear image was obtained. Using scanning data recorded as NIR photon signals, a grayscale image was constructed (Figure 2B). However, only a faint cell image could be obtained because the fluorescence spectra of dyes used to stain the cell sample were shorter than 800 nm. The photon number detected as NIR was as small as four photons (as median) for each pixel (Supplementary Figure S2B), although a TES is sensitive to the detection of NIR photons (Niwa et al., 2017). It has been reported that there are NIR fluorescence signals from cellular samples without labeling with any fluorescent dye, which is recognized as NIR auto-fluorescence (Paras et al., 2011). To confirm the potential of TES as a detector for NIR microscopy, further efforts should be made to observe much clearer NIR images associated with auto-fluorescence. By applying NIR fluorescent dyes, it is also possible to increase the labeling of dyes for cell imaging. Further progress of CLSM with TES is desired for NIR cell imaging.

In this report, photon energy information was transferred into four color-channel-distinguished signals to provide RGB and NIR images. Because the energy resolution Δ*E* of the TES used in this study was 0.1 eV, the wavelength resolution was approximately from 50 to 150 nm in the visible range. Thus, the detected fluorescence emission of the dyes partially overlapped each other, which could cause incorrect photon discrimination to the neighboring color channels. As shown in Figure 2A, however, three fluorescent dyes could be distinguished from each other in different colors, and the spatial distribution of each dye could be observed. If the spectra of fluorescent dyes are much closer and overlapped each other more, the overlap might deteriorate the RGB color quality. Even in this case, spectral overlapping can be statistically determined and compensated by increasing the number of color channels for TES detection (Nakajima et al., 2005). Here, the spectral information of each fluorescent dye is necessary, which can be obtained, for example, using the samples stained with each single fluorescent dye.

In Figure 2, the total duration time was 66 min, where the detection by TES took 26 min because the exposure time was 40 ms for each 200 × 200 pixel, and the resulting 40 min was associated with 0.4 μm scanning steps. This scanning condition differs slightly from that in Figure 3, in which the exposure time is 20 ms and the scanning step of 0.25 μm. In this condition for Figure 3, the total duration was 48 min, where detection via both TES or PMT took 13 min, and the remaining time was 35 min. Although the scanning steps were reduced from 0.4 to 0.25 μm, the scanning duration only reduced from 40 to 35 min, because the scanning duration mainly comprises data processing time behind stage movement. Therefore, the scanning duration can be significantly reduced by optimizing computer programs for data processing, resulting in a reduction in the total duration for capturing an image. Reducing the exposure time and scanning step numbers can also contribute to reducing the total duration of image capture. To reduce the exposure time, the photodetector must be more efficient for detection, which can be achieved by improving the optical absorption of the TES coatings. To reduce the number of scanning steps, the use of multiple photodetector elements contributes to reduction of scanning area. Thus, constructing an array-type TES device with a high detection efficiency will increase the demand.

### Simultaneous Detection of Blue, Green, and Red Fluorescent Dyes

As this report aims to prove the concept of wide-band spectral photon counting imaging using a TES to provide a full-color image in a single scan, the sample should emit fluorescence signals as wide as from blue to red. Test slides of cells were stained with three fluorescent dyes (DAPI, Alexa 488, MitoTracker). Most photodetectors cannot distinguish the incident photon energy; they only provide grayscale images. For example, CLSM using a PMT requires three individual scans for each fluorescent dye (Figures 3A–C). A pseudo-color image was developed by merging the three images digitally (Figure 3D). Controversially, using TES, RGB color images could be obtained even with single-laser irradiation (Figures 3E,F). It is notable that, without irradiating the 532 nm laser to excite MitoTracker, a remarkable fluorescent signal from MitoTracker was emitted by irradiating with the 405 nm laser (Figures 3A,E) and 488 nm laser (Figures 3B,F), because these wavelengths could be partially absorbed by and excite MitoTracker. Using TES, fluorescence emission from MitoTracker was detected as a red color and distinguished from DAPI (Figure 3E) and Alexa 488 (Figure 3F). When using PMT, however, MitoTracker was identically detected with, and could not be distinguished from, DAPI (Figure 3A) or Alexa 488 (Figure 3B). Therefore, by combining two color lasers of 405 and 488 nm and irradiating the cells simultaneously, all three fluorescent dyes could be excited to obtain an RGB color image in only a single scan (Figure 3G).

Because conventional optics based on dichroic mirrors are designed to separate shorter wavelengths of excitation light and longer wavelengths of fluorescence emission light, the excitation wavelength must be shorter than the fluorescence emission wavelengths. Therefore, a 405 nm laser was used to excite DAPI, whereas it could not excite Alexa 488 and MitoTracker efficiently. Therefore, an additional 488 nm laser was employed to excite longer-wavelength fluorescent dyes. Generally, a long-pass dichroic mirror at 425 nm is not appropriate for the 488 nm excitation laser to be removed. To solve this problem, we applied an imperfection of the dichroic mirror that retained 2.36% reflectance at 488 nm. Consequently, the 488 nm excitation laser was slightly reflected toward the objective and irradiated to the sample plane. Before detection of fluorescence emission, excitation lasers of 405 and 488 nm were removed using a long-pass filter (cut off 450 nm) and notch filter (at 488 nm), respectively. Thus, fluorescence signals from all three fluorescent dyes (DAPI, Alexa 488, MitoTracker) were successfully detected to provide a full-color image using two excitation lasers at 405 and 488 nm.

### Few-Photon Wide-band Imaging

To compare the detection efficiency between a TES and PMT, photon-counting signals were investigated from images in which the same sample was captured under the same irradiation and scanning conditions (Figure 4). The photon counting signals as medians by the TES for blue (*S*_B_), green (*S*_G_), red (*S*_R_), and NIR (*S*_NIR_) were 5, 53, 59, and 12, respectively, obtained from the area of cell presence in the images (Figures 4A,B). The photon-counting signal by the PMT was 32 (*S*_PMT_) (Figure 4C). These signal values for the TES and PMT were comparable, but it is notable that *S*_PMT_ is the sum of all fluorescence color signals. Nevertheless, *S*_PMT_ = 32 is remarkably less than the individual *S*_G_ = 53 and *S*_R_ = 59. Background signals from the empty area in the images were as small as 0–2 counts by both, the TES and PMT. These results indicate that TES can act as a spectral photon-counting detector for fluorescence cell imaging.

The photon counting numbers detected were in the order of 10^−17^ J. Because the exposure time for each pixel was 20 m, the energy levels were in the order of 10^−16^ W. Although the TES was less efficient in detecting shorter wavelength range photons (Niwa et al., 2017), the efficiency can be improved by optimizing the optical absorption in TES coatings. Furthermore, as the microscope optics reported were our original setup and quite primitive, there remain places for optimization. Commercially available high-end CLSM is also appropriate for employing a TES as a photodetector.

Although the detection efficiency of the PMT is high in the shorter wavelength region, the TES we used was not (Niwa et al., 2017). Nevertheless, sufficiently strong signals could be detected by the TES to depict cellular images. By optimizing the detection efficiency of the TES for a shorter wavelength region, the potential of CLSM using a TES could be improved.

Overall, the excitation irradiation power could be reduced to 100 nW to capture cell imaging, even using our primitive confocal optics with the TES. The excitation laser can be further reduced by improving the optics as well as the TES fabrication, suggesting the potential of the TES for use in cell-imaging applications as ultra-sensitive and wide-band wavelength spectral photodetectors.

## Conclusion

CLSM optics were constructed and tested for proof of concept using TES as a photodetector for few-photon spectral confocal microscopy imaging of cell samples. Using this microscope, observations of fluorescent-labeled cell samples were used to obtain a full-color image, as well as an IR image, by a single scan. Despite the preliminary observation, excitation laser irradiation at 100 nW was effective in providing cell image data. Thus, it was proved that TES is a potent photodetector for few-photon spectral confocal cell imaging.

## Data Availability

The original contributions presented in the study are included in the article/Supplementary Material, further inquiries can be directed to the corresponding author.
