# Supplementary material for: Few-Photon Spectral Confocal Microscopy for Cell Imaging Using Superconducting Transition Edge Sensor
Source: Front Bioeng Biotechnol. 2021 Dec 15;9:789709. doi: 10.3389/fbioe.2021.789709 (PMC8715037; doi:10.3389/fbioe.2021.789709)
Supplement: Supplementary file 1 [file DataSheet1.docx]

# Supplementary Material


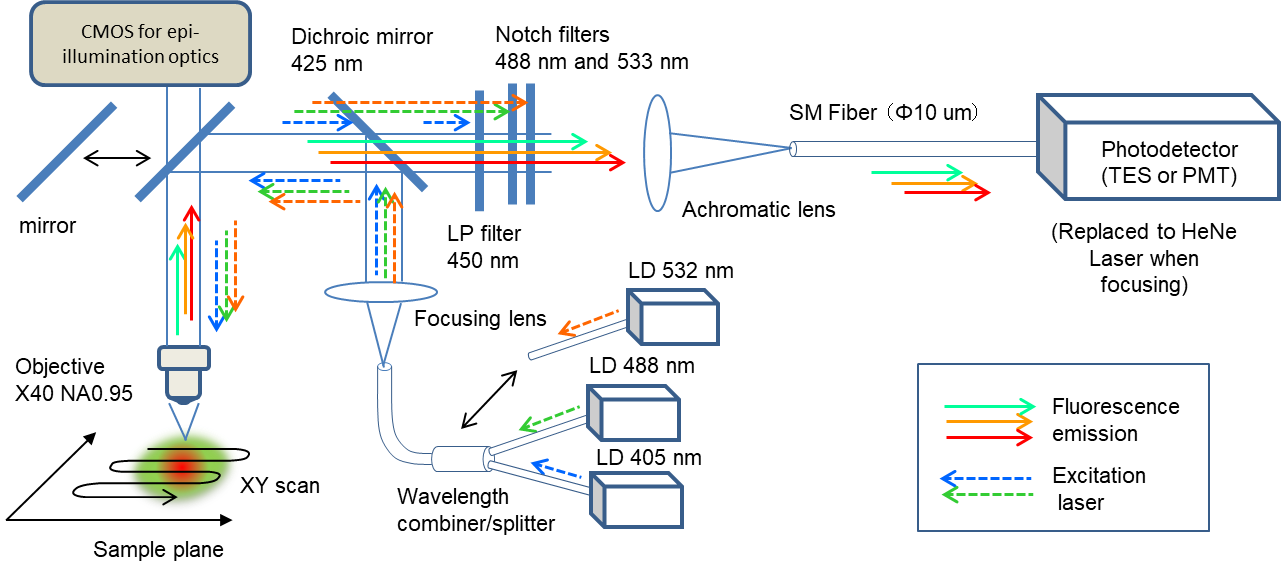


Supplementary Figure 1. CLSM optics set-up to compare the TES with the PMT. A laser diode at 532 nm and a notch filters before the photodetector were added to the set up described in Fig. 1. Individual excitation lasers as well as combined lasers at 405 and 488 nm can be introduced.


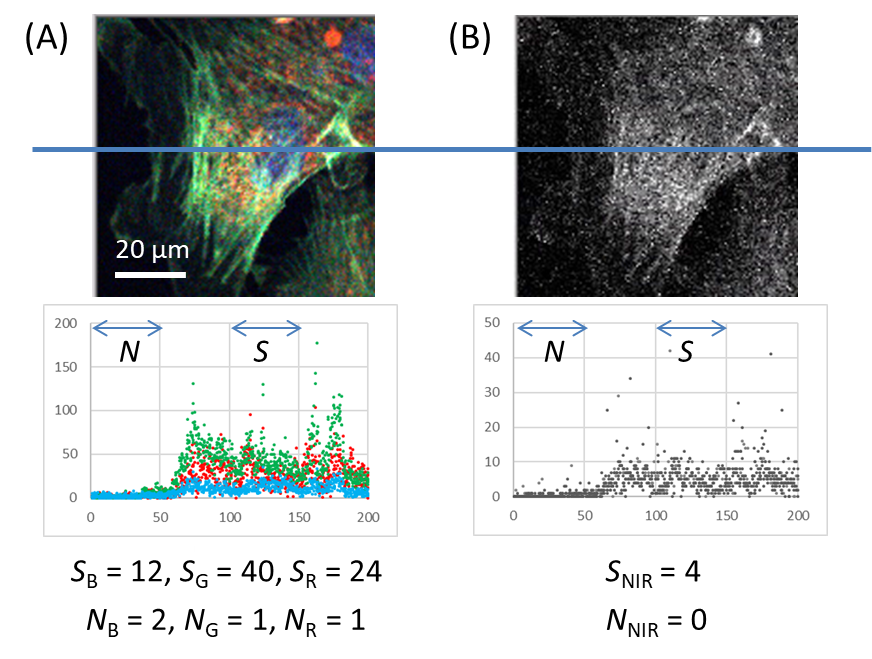


Supplementary Figure 2. Histograms of photon counting signals detected using the TES at the transection line (including five pixels width) on the images and signal (*S*) and background noise (*N*) intensity as medians of the areas indicated by arrows. **(A**) RGB image data, identical to Fig. 2A. **(B)** NIR image data, identical to Fig. 2B.
